# Dapagliflozin Mitigated Elevated Disomic and Diploid Sperm in a Mouse Model of Diabetes and Recover the Disrupted *Ogg1*, *Parp1*, and *P53* Gene Expression

**DOI:** 10.3390/biomedicines11112980

**Published:** 2023-11-06

**Authors:** Norah A. Albekairi, Mohammed A. Al-Hamamah, Ali A. Alshamrani, Mohamed S. M. Attia, Ahmed Nadeem, Mushtaq A. Ansari, Sheikh F. Ahmad, Saleh A. Bakheet, Sabry M. Attia

**Affiliations:** Department of Pharmacology and Toxicology, College of Pharmacy, King Saud University, Riyadh 11451, Saudi Arabia; nalbekairi@ksu.edu.sa (N.A.A.); mohammad_9119@hotmail.com (M.A.A.-H.); aaalshamrani@ksu.edu.sa (A.A.A.); mohamed.sabre2023@gmail.com (M.S.M.A.); muansari@ksu.edu.sa (M.A.A.); sbakheet@ksu.edu.sa (S.A.B.)

**Keywords:** hyperglycemia, SGLT2 inhibitors, numerical chromosomal aberrations, birth defects

## Abstract

Increases in numerical chromosomal syndromes were observed in children of diabetic mothers. However, the effects of diabetes on male reproduction, specifically numerical chromosomal aberrations (aneuploidy), have not been studied. Furthermore, despite the increasing use of dapagliflozin for diabetes treatment, no data exists on its ability to affect aneuploidy levels in germ cells. Thus, our investigation aimed to evaluate the effects of diabetes on spontaneous sperm aneuploidy and whether treatment with dapagliflozin influences the frequency of aneuploidy in the sperm of an experimental diabetic animal model. Our findings show that dapagliflozin has no aneugenic effects on the meiotic stages of spermatogenesis. In contrast, diabetes raised the frequency of aneuploidy, and dapagliflozin administration decreased the elevated levels of disomic and diploid sperm. The level of oxidative stress was markedly increased in diabetic mice, but were reduced by dapagliflozin treatment. Furthermore, the expression of some of DNA repair genes was disrupted in diabetic animals, whereas dapagliflozin therapy restored these disruptions and significantly enhanced DNA repair. Thus, dapagliflozin may effectively ameliorate diabetes-induced aneugenic effects on male meiosis and treating diabetic patients with dapagliflozin may effectively mitigate the transmission of diabetes-induced chromosomal defects to offspring.

## 1. Introduction

Diabetes is one of the most prevalent metabolic disorders that threaten human health, affecting more than 500 million adults, and its prevalence is expected to increase in the future [1]. Hyperglycemia is the most prevalent symptom of diabetes, which results in a decrease in insulin production in type 1 diabetes or full resistance or poor insulin secretion in type 2 diabetes [2]. Diabetes is currently one of the most serious public health concerns because of the consequences of prolonged hyperglycemia, which results in nephropathy, cardiovascular disease, neuropathy, retinopathy, and male and female reproductive injuries. Diabetes is becoming more common among younger people and some researchers have linked it to decreased birth and fertility rates [3,4]. Male gonads are functionally defined by continuous sperm generation with high energy requirements and high metabolic activities of male germinal cells, making them particularly susceptible to external and internal physiological disturbances [5]. Seminal insulin concentrations are often higher than serum concentrations, and defects in insulin secretion can alter gonadal function. Numerous studies have found that diabetes impairs reproductive function and pregnancy outcomes; however, the exact mechanism remains to be clarified [5,6,7].

The role of diabetes in the etiology of chromosomal defects has rarely been investigated; however, some women with gestational diabetes show underlying biochemical alterations that drive non-disjunction and the development of chromosomal defects. The prevalence of chromosomal abnormalities in the offspring of women with gestational diabetes is as high as that reported in the offspring of healthy women [8]. These defects are mostly numerical sex chromosomal anomalies. Because the male gamete accounts for half of the genetic makeup of the embryo, male factors are also important for embryo chromosomes. Numerous studies have shown that diabetes decreases sperm concentration and motility and increases aberrant sperm morphology in diabetic male humans and animals [5,6,7].

Chromosomal aberrations are a prevalent cause of infertility in humans and both structural and numerical abnormalities are main contributors to congenital deformities, miscarriages, retardations, behavior abnormalities, and perinatal deaths [9,10]. Autosomal chromosome trisomy and sex chromosome aneuploidies are the most common congenital anomalies. In most cases, sex chromosomal aneuploidy is paternal in origin and develop during the process of spermatogenesis, whereas autosomal trisomies originate in maternal oocytes [11].

Several mechanisms underlying reproductive function impairment in diabetes have been hypothesized. Oxidative stress, which induces chromosomal damage in affected cells, is one of the most critical pathophysiological causes for diabetes [12]. Sperm cells are exposed to a variety of conditions, including oxidative stress, which can cause DNA damage or increase the proportion of aneuploid sperms, thereby compromising male fertility and pregnancy outcomes [13]. Increased oxidative stress in diabetes can impair Leydig cell function and lower testosterone synthesis; as a result, patients with diabetes have a lower reproductive capacity [13]. Indeed, elevated blood glucose levels require intensive antidiabetic therapy, and many individuals with diabetes will have children after being diagnosed with diabetes and consequently receive antidiabetic drugs, which may have an impact on their reproductive health [14].

Sodium-glucose cotransporter 2 (SGLT2) blockers are a new class of antidiabetic drugs that enhance blood glycemic control and have cardio-renal protections in individuals with and without diabetes [15]. SGLT2 inhibitors can lower blood sugar levels even in conditions where the insulin secretory ability is reduced. Because they enhance glucose urinary excretion in a hyperglycemic condition without influencing insulin secretions, these new classes have a low risk of hypoglycemia. Additionally, SGLT2 inhibitors, such as dapagliflozin, lower the risks of increasing diabetic renal occasions and cardiovascular disease [15]. This suggests that SGLT2 blockers preserve hypoglycemia-targeted renal and cardiovascular systems [16]. We hypothesized that SGLT2 inhibitors may have a favorable effect on sperm aneuploidy because numerical chromosomal aberrations occur in diabetes-induced hyperglycemic impairment. Furthermore, patients using anti-diabetic agents must be examined for chromosome abnormalities to avert medication aneugenicity. Potential sperm cell impacts are a regular section of this process and are regularly examined throughout repeated dose-toxicity assessments.

Despite the growing uses of dapagliflozin in diabetes, there is little information about its potential harmful impacts on germ cells, with contradictory data available [17,18,19]. Therefore, assessing the aneugenic potential of dapagliflozin in diabetes treatment is essential to gain a better understanding of germinal cell toxicities, which could play a role in the increasing genetic risks in individuals with dapagliflozin-treated diabetes and future generations. Thus, the current study aimed to investigate whether dapagliflozin treatment affects the frequency of disomic/diploid sperm and redox balance in the spermatozoa of diabetic animals. Streptozotocin-induced hyperglycemia in mice is a useful model for assessing the influence of diabetes on different organs, such as the male sex glands, because alterations in the reproductive system induced by streptozotocin treatment are caused by a reduction in insulin level rather than a direct effect of streptozotocin [20]. To assess the levels of disomy and diploidy in epididymal spermatozoa, fluorescence in situ hybridization (FISH) analysis with DNA labeled probes specific for chromosomes 8, X, and Y was performed. The levels of glutathione (GSH) and 8-hydroxy-deoxy-guanosine (8-OHdG), which are markers of redox imbalance, were quantified as potential causes of the beneficial effects of dapagliflozin. Additionally, the molecular mechanism(s) underlying these ameliorations were elucidated by quantifying the expression level of certain genes involved in chromosomal segregation using the real-time quantitative reverse transcription polymerase chain reaction (RT-qPCR).

## 2. Materials and Methods

### 2.1. Animals

Seven-weeks-old male C57BL/6J mice weighing 19 ± 3 g were acquired from King Saud University’s Experimental Animal Care Center. The NIH Guides for the Care and Use of Laboratory Animals was followed by a proposal approved by King Saud University’s Research Ethics Committee (KSUSE-2357). Animals were housed in well-ventilated rooms at 22 ± 3 °C, 45–65% humidity, and 12-h light-dark cycles with regular water and food accessible *ad libitum*. The animals were permitted to adapt in the cages (five mice per cage) during the study. Each control and treatment group had ten animals. 

### 2.2. Experimental Diabetes Inductions

Diabetes was induced by administering an intraperitoneal dose of freshly streptozotocin solution in 100 mM citrate solution (pH 4.5) at a daily dosage of 60 mg/kg/day for five successive days [21]. On day 5 of the experiment, the animals were supplied with tap water with 10% sucrose for two days to prevent early death. Blood glucose levels were quantified a week after streptozotocin injection using tail venous blood, and diabetic animals with blood glucose level > 300 mg/dL were identified and used in our investigation.

### 2.3. Drug Administration

Diabetic animals were administered 10 mg/kg dapagliflozin (Selleck Chemicals, Suffolk, UK) by oral gavage once daily for 14 days [22]. The solvent (0.5% carboxymethylcellulose) was administered to the control animals in similar volumes. The experiment also included two non-diabetic groups that were orally administered dapagliflozin or vehicle. According to Oakberg’s report on the timing of mouse spermatogenesis [23], where he determined that it takes 22 days for the cells to develop from the meiosis stage to epididymal sperm, epididymal sperm cells were collected 22 days following the last dose of dapagliflozin to evaluate the consequences of dapagliflozin-caused missegregation during the male meiotic germ cell stage. Colchicine was used as a positive control aneugen and was administered to six animals 22 days before isoflurane euthanasia at an intraperitoneal dose of 3 mg/kg [24].

### 2.4. Sperm FISH Analysis

**Figure 1:**
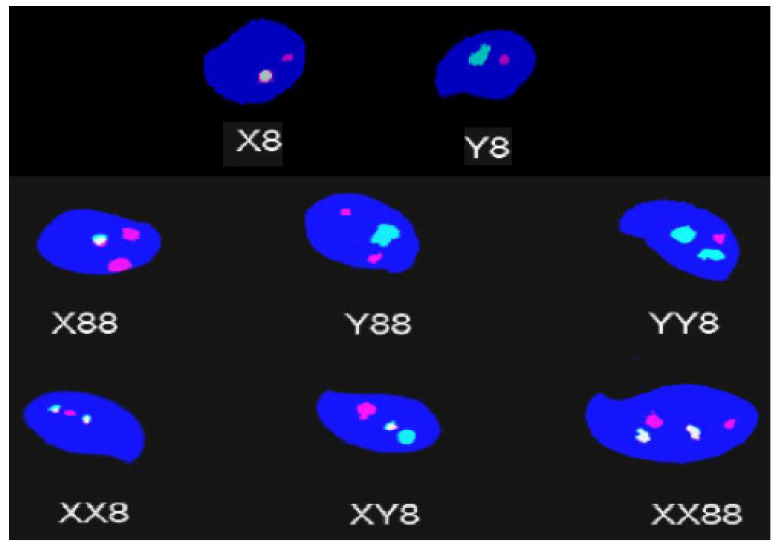
Representative images of different signals in sperm cells. Sperms were hybridized with labeled mouse-specific DNA probes for chromosomes 8 (red signals), Y (green signals), and X (yellow signals) [26].

The cauda epididymis of each animal were sampled and placed in tubes containing fetal bovine sera. Following 30 min of worming at 35 °C, epididymis tissues were taken out, and the spermatozoa were smeared on microscope glass slides and dried at room temperature or stored at −80 °C for later use. Using the nick translation system, digoxygenin-11-dUTP and biotin-16-dUTP were used to label mouse DNA probes specific for 8 (red) and Y (green) chromosomes, respectively. The chromosomal X DNA probe (yellow) was characterized with a mixture of digoxygenin-11-dUTP plus biotin-16-dUTP. Following denaturations, the labeled probes were incubated with sperm chromosomes on coded slides at 37 °C overnight in a moistened incubator. Biotinylated anti-streptavidin and anti-digoxygenin-FITC antibody were used to detect the hybridized DNA probes. Sperm nuclei were counterstained with DAPI and mounted with VectaShield^®^ solution, as described previously [25]. Fluorescent signals from 10,000 sperms per animal were examined. Sperms were classified as normal sperm (Y8 and X8), disomic sperm (Y88, XY8, and X88), or diploid sperm (YY88, XY88, and XX88) (Figure 1). A sperm with an extra chromosome resulting from chromosomal nondisjunction during meiosis is called a disomic sperm. In contrast, double disomic sperm caused by meiotic arrest is a diploid sperm with a duplicated set of all chromosomes. As previously explained, sperms were considered to have two domains of the same fluorescent color if both signals were (1) of identical intensity, (2) separated by at least half the diameter of one domain, and (3) not diffuse, regular in shape, and found inside the sperm head [26]. Disomic and diploid sperm are expressed in figures as percentages.

### 2.5. Markers of Redox Imbalance

To study the influence of dapagliflozin on redox imbalance caused by hyperglycemia, stored sperms were thawed and 8-OHdG, a common marker of oxidative damage to DNA molecule, and GSH levels were measured. The FitAmp DNA extraction kits (Epigentek, Farmingdale, NY, USA) was used to extract sperm DNA, then Dnase-1 (1 U/g DNA) was added to the isolated DNA before testing for 8-OHdG levels using the Bioxytech 8-OHdG Kits (OXIS Health Products, Portland, OR, USA) as reported earlier [27]. The level of sperm GSH was quantified with 5,5′-dithiobis (2-nitrobenzoic acid) according to the earlier technique [28]. GSH level was quantified using a standard curves achieved from GSH standard solutions. Absorbance were quantified using spectrophotometry along with a blank (BMG LABTECH Ltd., Ortenberg, Germany).

### 2.6. Gene Expression Analysis

To investigate the influence of dapagliflozin on repair deficiency induced by hyperglycemia, stored sperms were thawed and RT-qPCR was applied to evaluate the expression of some repair genes involved in chromosomal segregation to elucidate the molecular mechanism(s) by which the dapagliflozin can reverse aneuploidy induction. Total RNA was isolated from the thawed sperm cells using the RNeasy Mini Kits (Qiagen, Valencia, CA, USA), based on the manufacturer’s recommendation. A Nanodrop spectrophotometer was used to measure RNA purity and quantity. As previously described [29], total RNA was reverse transcribed into cDNA using a high-capacity cDNA reverse transcription kit (Applied Biosystems, Waltham, MA, USA). The generated cDNA was added to SYBR Green PCR Master Mix before running on a 7500 RT-qPCR System. Primer sequences are shown in Table 1. After normalizing the results to the level of the GAPDH housekeeping gene, the relative mRNA expressions were quantified using the 2^−ΔΔCT^ formula [30]. 

### 2.7. Statistics

Variables are expressed as the mean ± standard error (SE). Data were tested for normality and homogeneity using GraphPad Prism before analysis by ANOVA with the Tukey–Kramer test for multiple comparisons or the Mann–Whitney *U* Test and Kruskal–Wallis with Dunn’s test for multiple comparisons. *p* values less than 0.05 were considered significant.

## 3. Results

### 3.1. Dapagliflozin Mitigated Diabetes-Induced Disomy and Diploidy

**Figure 2:**
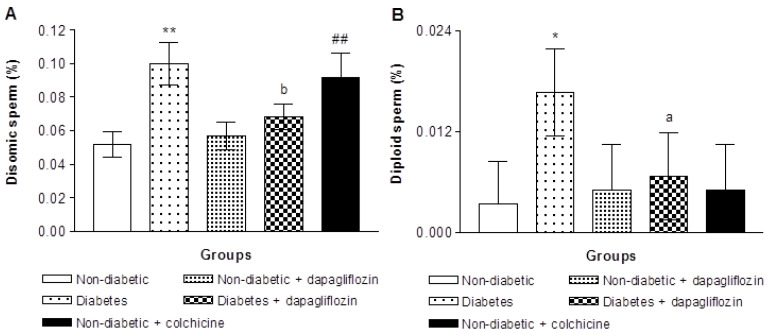
Incidences of disomy (**A**) and diploidy (**B**) in animals without and with dapagliflozin therapy or the positive control aneugen colchicine (mean ± SE). * *p* < 0.05, ** *p* < 0.01 vs. non-diabetic control animals (Kruskal–Wallis test). ^a^ *p* < 0.05, ^b^ *p* < 0.01 vs. untreated diabetic animals and ^##^ *p* < 0.01 vs. control non-diabetic animals (Mann–Whitney *U* Test).

To study the possible aneuploidogenic effects of dapagliflozin and diabetes on mouse sperm cells, the incidence of disomy and diploidy was estimated by a sperm-FISH assay using DNA labeled probes. Table 2 and Figure 2 show the results of sperm FISH assays. A total of ∼60,000 sperm were scored from 6 animals in each group, and 303,780 sperm were scored from all groups (Table 2). As expected, the ratio of the sex chromosomes was in the same range as the theoretical ratio (1:1 for Y- vs. X-carrying sperms). A total of 152,500 sperm bearing the Y chromosome and 151,280 sperm bearing the X chromosome were scored in all mice, and there were no significant differences in the Y/X ratio among the examined mice (Table 2). No substantial change in aneuploidy levels after repeated dapagliflozin treatments [disomic (0.056% against 0.051% in the control group) and diploidy (0.005% against 0.003% in the control group)] (*p* > 0.01, Table 2). This indicated that dapagliflozin had no aneugenic impacts on meiotic stage at this dosing regimen. 

Diabetes, on the other hand, increased the frequency of disomic sperm (0.10%) compared with that in the non-diabetic mice (0.051%) (Figure 2A, *p* < 0.01). Furthermore, the incidence of diploid sperm in diabetic animals was substantially higher than those from non-diabetic mice, indicating that full meiotic arrest had happened (0.016% against 0.003% for the control, Figure 2B). Treatment of diabetic animals with dapagliflozin for 14 days after diabetes inductions reduced the elevated levels of disomic spermatozoa (0.068% compared to 0.1%) and diploid sperm (0.006% compared to 0.016%). The frequency of disomic sperm, as produced by the positive control aneugen colchicine, exhibited a statistically significant increase of 1.78 times when compared to the control value of 0.051% (0.091%; *p* < 0.01). Nevertheless, colchicine did not increase the frequency of diploid sperm (0.005% vs. 0.003% in control, Table 2).

### 3.2. Dapagliflozin Abridged Diabetes-Caused Redox Imbalance 

**Figure 3:**
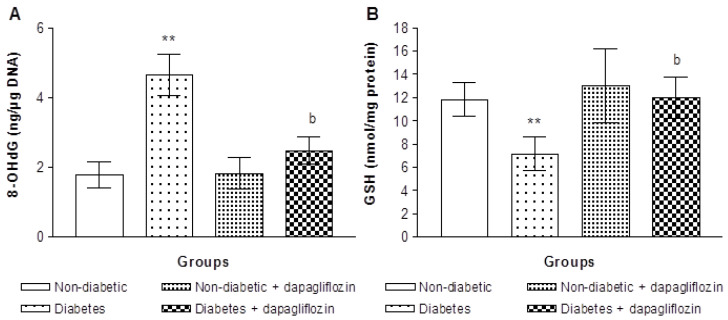
Sperm 8-hydroxydeoxyguanosine (8-OHdG, (**A**)) and reduced glutathione (GSH, (**B**)) levels in animals without and with dapagliflozin treatment (mean ± SE). ** *p* < 0.01 vs. non-diabetic control mice (ANOVA test). ^b^ *p* < 0.01 vs. diabetic mice (Student’s *t*-test).

The influence of dapagliflozin on diabetes-caused redox imbalance was evaluated by determining 8-OHdG and GSH levels in sperm cells. No significant alteration in 8-OHdG concentrations between dapagliflozin-treated animals and non-diabetic control mice, as shown in Figure 3A. Diabetic animals exhibited a significant rise in sperm 8-OHdG concentration compared to non-diabetic control animals. When diabetic animals were administered dapagliflozin for 14 days following diabetes inductions, there was a noticeable and significant decrease in the sperm levels of 8-OHdG produced by diabetic animals compared with those of untreated diabetic animals (*p* < 0.01). Figure 3B demonstrations the GSH content of sperm cells in animals treated with and without dapagliflozin. When comparing dapagliflozin-treated animals with control mice, we found no differences in sperm GSH content. In contrast, the GSH level in diabetic animals was considerably lesser than that in control animals (*p* < 0.01). Compared with untreated diabetic animals, treatment with dapagliflozin for 14 consecutive days after diabetes induction improved the reduced of sperm GSH levels.

### 3.3. Alterations in Gene Expression 

**Figure 4:**
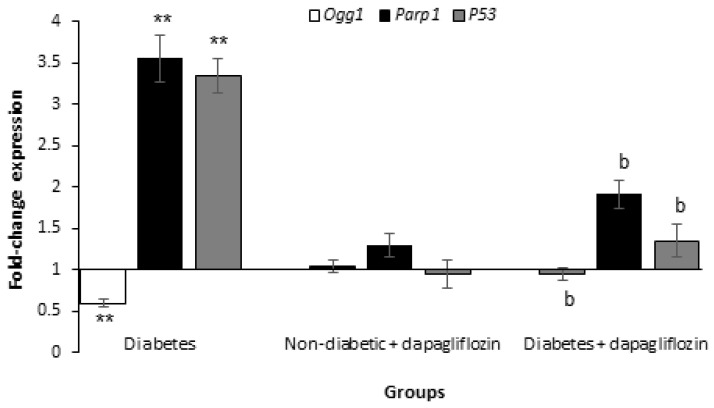
RT-qPCR study of *Ogg1*, *Parp1* and *P53* genes implicated in DNA repair in the sperm cells of animals with and without dapagliflozin treatment (mean ± SE). Fold change expression is shown relative to non-diabetic control mice set at 1.00. ** *p* < 0.01 vs. non-diabetic control mice (ANOVA test). ^b^ *p* < 0.01 vs. diabetic mice (Student’s *t*-test).

Changes in the relative gene expression of some repair genes involved in chromosomal segregation were evaluated in sperm cells using RT-qPCR to identify the molecular targets related to chromosomal aberrations in diabetic animals and to explore the possible mechanism(s) underlying the ameliorative influences of dapagliflozin. Dapagliflozin-treated non-diabetic mice did not demonstrate any substantial alterations in the expression of *Ogg1*, *Parp1* or *P53* genes (Figure 4). *Ogg1* expression was considerably lower in diabetic animals than in healthy control animals. However, *Parp1*, and *P53* expression increased in diabetic animals (*p* < 0.01), indicating altered DNA repair. In dapagliflozin-treated diabetic mice, the expression levels of the altered genes were significantly reversed, and *Ogg1*, *Parp1*, and *P53* levels in treated diabetic mice were nearly similar to healthy mice.

## 4. Discussion

DNA strand breaks are the most common genetic abnormalities identified in ejaculated human spermatozoa [31]. Hence, scientific emphasis has been placed on sperm DNA damage over the past two decades. In particular, the discovery that large amounts of sperm DNA strand breaks can be observed in the spermatozoa of diabetic men has aroused concerns about their reproductive functions and, more crucially, the health of their progeny [32]. Spermatozoa are highly specialized cells responsible for transferring an intact paternal genome to oocytes and successfully supporting embryonic development. Because glucose metabolism is a crucial component of spermatogenesis, diabetes can disrupt these processes. An increased prevalence of chromosomal defects has been observed in the children of diabetic mothers [8,33]. However, the effects of diabetes on male reproduction, particularly chromosomal complement aneuploidy, have received little attention.

The current study used the well-known spindle poison colchicine as a germ cell aneugen, which prevents the polymerization of tubulins. Although the incidence of disomic sperm was much greater in colchicine administrated group than in the non-diabetic control group, there was no appearance of diploid sperm, indicating that no complete meiotic arrest happened. These results are in line with previous findings published previously [26]. Dapagliflozin administration in non-diabetic animals did not increase the occurrence of aneuploid sperm. In fact, dapagliflozin and its metabolites were tested for their ability to cause clastogenicity in the somatic cells of female and male rats; up to the highest evaluable doses, dapagliflozin did not cause structural chromosomal aberrations [34]. These findings exclude aneuploidogenic and clastogenic effects of dapagliflozin in germinal cells (current study) and somatic cells [34], respectively.

Chromosomal aberrations are genetic features of several metabolic disorders, such as diabetes and obesity [35]. The incidence of diabetes among the parents of children with aneuploid sex chromosomes is higher than that among the parents of the general population [33,36,37]. In the current investigation, we observed that diabetes caused a rise in the frequency of aneuploid sperms, which was significantly higher in both disomic and diploid aberrations. These data agree with earlier findings of increased incidence of numerical chromosomal disorders in the children of parents with diabetes and support the theory that parents who develop diabetes may have underlying biochemical alterations that cause non-disjunction and the development of chromosomal defects [8,33]. 

This in vivo tendency was comparable to that observed in earlier chromosomal investigations of murine embryonic blastocysts cultured under diabetic conditions [38]. Blastocysts from the glucose-exposed group had greater rates of chromosomal abnormalities than those from the control group, particularly numerical abnormalities such as aneuploidy and polyploidy. These findings indicate that hyperglycemia may directly lead to chromosomal abnormalities in developing embryos, particularly aneuploidies, which may result in gene duplications or deletions. This chromosomal damage may interfere with organogenesis and developmental programs and contribute to diabetes-induced teratogenesis. In the present investigation, dapagliflozin therapy in diabetic mice decreased the overall incidence of disomic and diploid sperms in comparison to that in diabetic mice. Increased level of disomic and diploid occasions in sperm cells of diabetic mice are likely as a result of biochemical changes that cause non-disjunction and the development of chromosomal defects. Importantly, the frequency of aneuploid sperm in diabetic mice was higher than in control mice. Although the level is very low (0.10% for disomic sperm), sperm aneuploidy negatively influences reproduction outcomes. Even though it does not affect fertilization rates, increased sperm aneuploidy rates are linked to a greater rate of pregnancy failures. Nevertheless, some patients with increased sperm aneuploidy rates can still succeed in pregnancy, but with an elevated risk of generating aneuploid children [39].

Increasing evidence suggests that disturbances in the redox balance and development of diabetes are closely related. The occurrence of a disturbed redox balance supports various metabolic changes that cause oxidative damages to lipids, proteins and DNA, and subsequently to redox imbalance, which further worsens diabetes and causes secondary diseases, such as impairment of reproductive capability [7,40]. The relationship between diabetes and oxidative stress is bidirectional; oxidative stress may result in a response to metabolic changes linked with diabetes but has also been linked as a contributor to diabetes and its related complication [41]; this indicates that one of them can just as well initiate the other. Therefore, we tested whether the reduction in aneuploidy observed after dapagliflozin treatment also restored the redox balance in diabetic animals. Our data showed that the levels of 8-OHdG was higher in diabetic animals, which is in agreement with earlier results in diabetic individuals and experimental diabetic animals [42,43]. Conversely, dapagliflozin-treated diabetic mice had lower levels of 8-OHdG in their sperms.

Numerous studies have demonstrated depletion of endogenous GSH in specimens from patients with diabetes [44,45]. Additionally, GSH deficiency results in instabilities of the spermatozoa midpiece, which affects the morphology and motility of spermatozoa [46]; these alterations have an impact on male reproduction. In line with previous findings in other investigational diabetic mouse model [47], lower sperm GSH level was found in diabetic mice in our study. These results show that diabetic mice have a reduced antioxidant defense system and higher redox imbalance, which trigger aneuploidy if the antioxidant defense mechanism fails. In contrast, diabetic mice treated with dapagliflozin showed restored GSH levels. Thus, dapagliflozin may have a beneficial effect on cellular defenses against diabetes-induced aneuploidy by reducing oxidative stress on DNA.

In our recent study [19], we have found that treatment of diabetic mice with dapagliflozin did not cause any significant harmful effects on testicular tissues. Moreover, diabetic animals showed clear changes in their testes, including elevated sperm DNA damage, elevated spermatocyte chromosome abnormalities, reduced sperm motility and counts, elevated spermatozoa morphological abnormalities, and a higher redox imbalance than those in controls; nevertheless, dapagliflozin treatment completely reversed these changes [19]. Our findings support the impact of dapagliflozin on reducing oxidative stress on DNA and mitigating the detrimental impacts on the male reproductive system in diabetic mice, as reported earlier [18]. On the other hand, sperm cells with abnormal shapes were found in higher numbers in diabetic rats that were given dapagliflozin compared to the control rats. In other words, dapagliflozin-induced damage to sperm morphology was independent of the effect of diabetes. While the improvements occurring in all sperm and testicular measured parameters are thought to result from antioxidant effects, deterioration in sperm morphology suggests that another mechanism may induce these morphological impairments [17]. 

DNA repair efficiency is reduced in individuals with diabetes compared to normal controls, and gene expression analyses have revealed the suppression of DNA repair genes in individuals with diabetes [48,49]. Diabetes has a complicated polygenic nature, with most loci increasing diabetic risk by directly affecting insulin secretion and a minority acting by decreasing insulin action [50]. Polymorphisms in DNA repair genes may influence repair capacity, predisposition to diabetes, and associated germ cell consequences. Furthermore, the risk of diabetes is significantly increased by polymorphisms in Rad18 and Xpd repair genes [51]. Xrcc1 repair gene polymorphisms are also positively associated with an increased predisposition to diabetes and its complications [52], adding to the evidence for defective repair genes in diabetes.

The expression of *Ogg1*, *Parp1*, and *P53* repair genes, which are available in our laboratory and have a significant role in chromosomal segregation, was examined in diabetic animals with and without dapagliflozin therapy to better understand their repair capabilities. Compared with non-diabetic control mice, *Ogg1* expression was significantly downregulated, and *Parp1*, and *P53* repair expression were upregulated in diabetic mice. Furthermore, dapagliflozin therapy restored gene disruptions in diabetic animals and improved these values to those found in non-diabetic control animals. The disruption of DNA repair genes confirms that defective repair is responsible for the increase of chromosomal aberrations detected in diabetic mice, consistent with previous investigations revealing disturbance of the DNA repair pathway in diabetes [48,49,53]. 

Ogg1 is a DNA repair enzyme recruited to oxidative stress-caused DNA damage by identifying and removing 8-OHdG from DNA molecule and encouraging a well-preserved base excision repair process [54]. Increased sperm redox imbalance in diabetic mice may be related to a significant decrease in *Ogg1* expressions. Under oxidative stress, Ogg1 is most likely necessary for proper cell cycle progressions and cell divisions. Ogg1 deficiency enhances cell susceptibility to oxidants, resulting in cell cycle arrest, complemented with centrosome amplification, and multipolar spindle formation [55], pulling chromosomes in numerous directions during segregation, which may be responsible for the development of chromosomal aneuploidy.

Parp1 functions as a genomic DNA breakage sensor. It has been demonstrated that overactivation of Parp1 contributes to the pathogenesis of numerous disorders linked with oxidative stress, which is known to play a critical role in male reproduction [56]. Additionally, Parp1 activation plays a significant role in the pathology of various diabetic complications, and its inhibition protects against these complications [57]. Parp1 inhibition restores the cellular response to DNA repair and reduces oxidative damage. Importantly, after dapagliflozin treatment, the 8-OHdG level in diabetic mice was significantly reduced, indicating that inhibiting Parp1 elevation may alleviate diabetic complications by reducing cellular oxidative chromosomal damage. P53 is a multifunctional regulatory protein that interacts with multiple important pathways, either directly or indirectly. It regulates the cell cycle, directs pathways for DNA repair and apoptosis, and controls multiple cell-cycle checkpoints. Numerous studies have found P53 to be involved in spermatogenesis, and increased sperm aneuploidy in male carriers of sperm P53 mutations has been reported [58,59]. *P53* expression was considerably higher in diabetic animals than in control healthy animals, demonstrating increased spermatogenesis disturbances in diabetic mice, which was reversed by dapagliflozin treatment. Normalization of the disrupted *Ogg1*, *Parp1*, and *P53* gene expression observed in diabetic mice after dapagliflozin treatment may be a secondary effect, while another molecular pathway involved in chromosome segregation has a direct role in the alleviation of sperm aneuploidy.

## 5. Conclusions

Our findings indicated that dapagliflozin treatment did not alter chromosomal numbers in mouse sperm cells, demonstrating that dapagliflozin had no aneuploidogenic influence on the meiotic stages of spermatogenesis. Spontaneous aneuploidy and disturbance in redox balance and repair disturbance were significantly higher in diabetic mice than in non-diabetic control animals. When diabetic mice were treated with dapagliflozin, the percentage of aneuploid sperm was significantly lower than that in untreated diabetic mice. The increased levels of aneuploid sperms and disrupted gene expressions observed in diabetic mice were significantly alleviated by dapagliflozin treatment through the restoration of the redox imbalance, which appear to be important mechanisms of amelioration. 

Diabetes has various etiologies, and the chemically-induced hyperglycemia in animals can’t represent outcomes in all cases. So, more research should be done on diabetic people and diabetic transgenic animals. Sperm cells are a challenging cell type for the analysis of RNA at the single-cell level, as sperm cells differ from typical somatic cells in some features; sperm cells are transcriptionally restricted, retaining only a small amount of RNA per cell (≈50 fg), which exists in a fragmented or partially degraded state, as transcription ceases during the spermatid stage of spermatogenesis. Additionally, sperm cells exhibit highly compact chromatin and have a minimal cytoplasm that could affect the levels of gene expression and redox balance. Therefore, other master regulator genes involved in cell cycle progression and chromosomal segregation should also be quantified at the mRNA and protein levels in testicular tissues, which is the limitation of our study. This could be interesting and give us more information on how dapagliflozin affects testicle complications caused by hyperglycemia. Taken together, dapagliflozin is a non-aneugenic antidiabetic agent that may protect against aneuploidy caused by diabetes, suggesting its salubrious importance in patients with diabetes, especially in those who become parents.

## Figures and Tables

**Table 1:** List of the primers.

Gene	Forward Primer	Reverse Primer
*Parp1*	5′-GGAAAGGGATCTACTTTGCCG-3′	5′-TCGGGTCTCCCTGAGATGTG-3′
*Ogg1*	5′-GATTGGACAGTGCCGTAA-3′	5′-GGAAGTGGGAGTCTACAG-3′
*P53*	5′-CACAGCGTGGTGGTACCTTA-3′	5′-TCTTCTGTACGGCGGTCTCT-3′
*GAPDH*	5′-TGCCGCCTGGAGAAACC-3′	5′-TGAAGTCGCAGGAGACAACC-3′

*Parp1 =* Poly [ADP-ribose] polymerase 1, *Ogg1* = 8-Oxoguanine glycosylase, *P53* = Tumor protein, *GAPDH* = Glyceraldehyde 3-phosphate dehydrogenase.

**Table 2:** Mouse sperm-FISH results for chromosomes 8, X and Y in cauda epididymis of animals with and without dapagliflozin treatment or the positive control aneugen colchicine (3 mg/kg) (mean ± SE).

	Non-Diabetic	Non-Diabetic + Dapagliflozin	Diabetes	Diabetes + Dapagliflozin	Non-Diabetic + Colchicine
No. of mice	6	6	6	6	6
Total sperm scored	60,580	61,100	60,820	60,280	61,000
Y bearing sperm	30,260	30,499	30,841	29,991	30,909
X bearing sperm	30,320	30,601	29,979	30,289	30,091
Y/X ratio	1.001	1.003	0.972	1.009	0.973
**Disomy**					
X-X-8	11	10	15	11	14
Y-Y-8	7	6	13	10	13
X-Y-8	2	3	9	7	8
X-8-8	4	6	10	5	10
Y-8-8	7	9	13	8	10
Total	31	34	60	41	55
% Disomy ± SD	0.051 ± 0.003	0.056 ± 0.003	0.10 ± 0.005 **	0.068 ± 0.003 ^b^	0.091 ± 0.006 ^##^
**Diploidy**					
X-Y-8-8	1	1	3	1	1
X-X-8-8	1	1	3	2	1
Y-Y-8-8	0	1	4	1	1
Total	2	3	10	4	3
% Diploidy ± SD	0.0033 ± 0.002	0.005 ± 0.002	0.0166 ± 0.002 *	0.006 ± 0.002 ^a^	0.005 ± 0.002

* *p* < 0.05, ** *p* < 0.01 vs. non-diabetic control animals (Kruskal–Wallis test). ^a^ *p* < 0.05, ^b^ *p* < 0.01 vs. untreated diabetic animals and ^##^ *p* < 0.01 vs. control non-diabetic animals (Mann–Whitney *U* Test).

## Data Availability

The authors confirm that all data underlying the findings are fully available without restriction.
